# Association between serum iron status and the risk of five bone and joint-related diseases: a Mendelian randomization analysis

**DOI:** 10.3389/fendo.2024.1364375

**Published:** 2024-09-13

**Authors:** Xiaolei Wang, Linjing Qiu, Zepei Yang, Changjiang Wu, Wenying Xie, Jing Zhang, Wenhui Li, Wangyang Li, Yanbin Gao, Taojing Zhang

**Affiliations:** ^1^ Department of Endocrinology, Dongfang Hospital, Beijing University of Chinese Medicine, Beijing, China; ^2^ Graduate School, Beijing University of Chinese Medicine, Beijing, China; ^3^ Beijing Key Laboratory of Traditional Chinese Medicine (TCM), Collateral Disease Theory Research, School of Traditional Chinese Medicine, Capital Medical University, Beijing, China

**Keywords:** bone and joint-related diseases, Mendelian randomization, iron status, genome wide association studies, genetics

## Abstract

**Background:**

According to reports, iron status has been associated with the risk of bone and joint-related diseases. However, the exact role of iron status in the development of these conditions remains uncertain.

**Method:**

We obtained genetic data on iron status, specifically serum iron, ferritin, transferrin saturation (TSAT), and transferrin, as well as data on five common bone and joint-related diseases (osteoarthritis, osteoporosis, rheumatoid arthritis [RA], ankylosing spondylitis [AS], and gout) from independent genome-wide association studies involving individuals of European ancestry. Our primary approach for causal estimation utilized the inverse variance weighted (IVW) method. To ensure the reliability of our findings, we applied complementary sensitivity analysis and conducted reverse causal analysis.

**Result:**

Using the IVW method, we revealed a positive causal relationship between ferritin levels and the risk of osteoarthritis (OR [95% CI], 1.0114 [1.0021-1.0207]). Besides, we identified a protective causal relationship between serum iron levels and TSAT levels in the risk of RA (OR [95% CI] values of serum iron and TSAT were 0.9987 [0.9973-0.9999] and 0.9977 [0.9966-0.9987], respectively). Furthermore, we found a positive causal relationship between serum iron levels and the risk of AS (OR [95% CI], 1.0015 [1.0005-1.0026]). Regarding gout, both serum iron and TSAT showed a positive causal relationship (OR [95% CI] values of 1.3357 [1.0915-1.6345] and 1.2316 [1.0666-1.4221] for serum iron and TSAT, respectively), while transferrin exhibited a protective causal relationship (OR [95% CI], 0.8563 [0.7802-0.9399]). Additionally, our reverse causal analysis revealed a negative correlation between RA and ferritin and TSAT levels (OR [95% CI] values of serum iron and TSAT were 0.0407 [0.0034-0.4814] and 0.0049 [0.0002-0.1454], respectively), along with a positive correlation with transferrin (OR [95% CI], 853.7592 [20.7108-35194.4325]). To ensure the validity of our findings, we replicated the results through sensitivity analysis during the validation process.

**Conclusion:**

Our study demonstrated a significant correlation between iron status and bone and joint-related diseases.

## 1 Introduction

Iron metabolism refers to a series of processes involved in the absorption, transportation, storage, utilization and excretion of iron within an organism. Disruptions in these metabolic processes can result in abnormal iron concentration and distribution in the body or cells, leading to various diseases, including bone and joint-related conditions such as osteoporosis, osteoarthritis, gout, and rheumatoid arthritis (RA). However, the existing epidemiological evidence on the impact of iron status on major bone and joint-related diseases remains inconclusive. For instance, Cheng et al. reported a negative correlation between serum iron levels and gout (1). However, a cohort study demonstrated a positive correlation between serum ferritin and gout, while serum iron showed no association (2). Furthermore, some studies have indicated no relationship between iron intake and RA (3), whereas others have reported a negative correlation between serum iron and RA (4). Consequently, it remains uncertain whether iron status influences the risk of developing bone and joint-related diseases.

To address this uncertainty, Mendelian randomization (MR) presents a more reliable research design than traditional observational studies for investigating the causal effects of exposure to diseases (5). By utilizing randomly assigned genetic predictive factors, similar to randomization in randomized controlled trials (RCTs), MR minimizes potential confounding variables (5). Additionally, MR studies are more often consistent with RCTs than traditional observational studies (6, 7). An increasing number of Mendelian studies have explored the causal relationship between iron status and bone and joint diseases. Yuan S et al. found that high iron status was positively correlated with gout and negatively correlated with RA through Mendelian studies (4). Xu J et al. provided evidence for a causal relationship between iron status and OA through Mendelian studies, providing novel insights to the genetic research of OA (8). However, Mendelian studies on the association between iron status and bone diseases are relatively limited.

In our comprehensive evaluation of the role of iron status in bone and joint-related diseases, we employed a genetic tool derived from a genome-wide association study (GWAS) of iron status biomarkers (9). However, due to the lack of a comprehensive GWAS database specifically related to bone and joint-related diseases, it is not feasible to address all genetic disorders associated with the skeletal system. Therefore, we focused our investigation on five prevalent bone and joint-related diseases (osteoarthritis, osteoporosis, RA, ankylosing spondylitis [AS], and gout) for which high-quality GWAS data are publicly available. In this study, our aim was to examine the causal relationship between iron status and these five common bone and joint-related diseases through MR analyses.

## 2 Methods

This study was in accordance with Strengthening the Reporting of Observational Studies in Epidemiology-Mendelian Randomization (STROBE-MR) guideline (10) (**Supplementary Table S1**).

### 2.1 Study design

**Figure 1:**
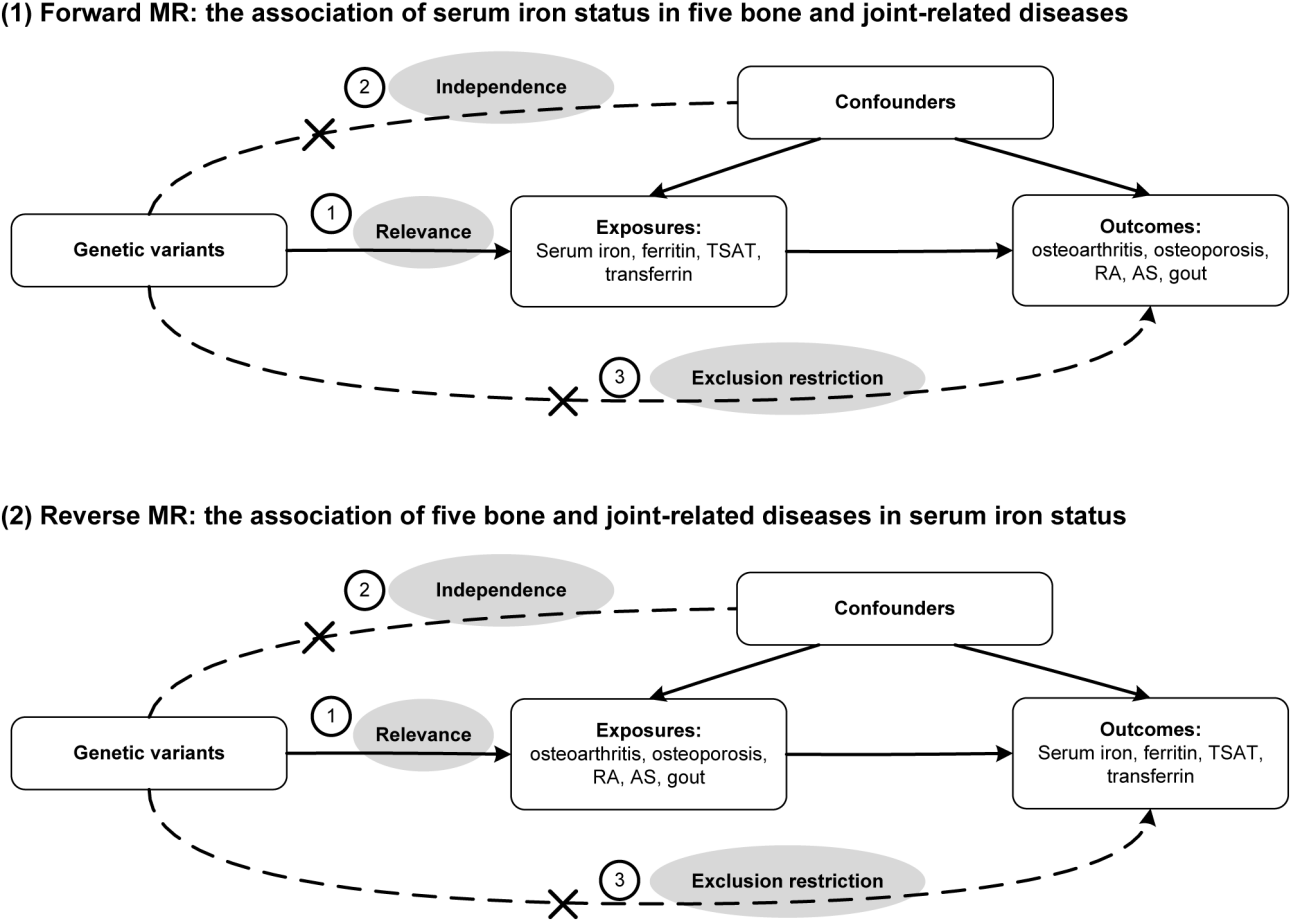
Directed acyclic graph of this bidirectional Mendelian randomization (MR) study. TSAT, transferrin saturation; RA, rheumatoid arthritis; AS, ankylosing spondylitis.

The research design is presented in **Figure 1**, incorporating four iron status biomarkers as exposure factors and five bone and joint-related diseases (osteoporosis, osteoarthritis, RA, AS, and gout) as outcomes. GWAS data for both exposure and outcome groups were collected. These data were then utilized for MR analysis to investigate the causal impact of iron status on different bone and joint-related diseases. MR relies on three assumptions: correlation, independence, and exclusion restrictions. Correlation refers to the ability of genetic tools, such as single nucleotide polymorphisms (SNPs), to predict exposure. Independence ensures that genetic tools remain separate and independent from confounding factors encountered in the results. Exclusion restrictions indicate that genetic tools are not associated with exposure outcomes, thus avoiding horizontal pleiotropy or selection bias.

### 2.2 Data sources

#### 2.2.1 Biomarkers of iron status

Genetic data pertaining to serum iron status was sourced from the Open GWAS project of the Integrated Epidemiological Unit (IEU) (https://gwas.mrcieu.ac.uk/). Four biomarkers of iron status, namely serum iron, ferritin, transferrin saturation (TSAT), and transferrin, were included as markers representing distinct aspects of iron status, including iron storage (ferritin) and transport (serum iron, transferrin, and TSAT). The genetic data for iron status was derived from a GWAS (9) comprising 48,972 individuals of European descent. Within this GWAS, summary statistics of genome-wide allelic associations between SNP genotypes and iron status markers were obtained from 23,986 participants of European ancestry across 11 cohorts from 9 participating centers. Duplicate samples from an additional 24,986 individuals of European ancestry in 8 other cohorts were used to confirm significant and suggestive associations. GWAS testing, genotype imputation, and quality control procedures were independently conducted in each cohort (9). The additive model of allelic effects adjusted for age, principal component scores, gender-specific study covariates, and standardized residuals of the phenotype were employed to assess the association between genotypes, estimated SNPs and each iron phenotype (9).

#### 2.2.2 Bone and joint-related diseases

All the data utilized for bone and joint-related diseases in this study were sourced from publicly available GWAS studies. Specifically, for osteoporosis, the data were obtained from the IEU OpenGWAS project, including 1,976 cases and 461,034 controls within the European population. Osteoarthritis data were acquired from the same project, consisting of 38,472 cases and 424,461 controls from the European population. For RA, the data were sourced from the IEU OpenGWAS project, including 5,201 cases and 457,732 controls from the European population. AS data were also obtained from the IEU OpenGWAS project, including 1,296 cases and 461,637 controls from the European population. Lastly, gout data were extracted from a large GWAS meta-analysis involving 2,115 cases and 67,259 controls specifically from the European population (11). **Supplementary Table S2** displays the relevant publications associated with each dataset.

### 2.3 Statistical analyses

To assess the impact of genetic tools on exposure and outcomes, we compared the allele frequency (EAF). Variance (R^2^) and population F-statistic were estimated for each exposure to determine the extent explained by genetic tools. A higher F-statistic indicates a lower likelihood of weak instrument bias. The main analysis used inverse variance weighting (IVW) with multiplicative random effects (12, 13). A fixed-effects meta-analysis was performed on the IVW estimates for bone and joint-related diseases. However, the balance of pleiotropy hypothesis in IVW may be violated by ineffective instruments. Therefore, we assessed the heterogeneity of Wald ratio using I^2^, which is obtained by dividing the genetic variation of the result by the exposed genetic variation. A high I^2^ suggests the presence of ineffective instruments (14).

### 2.4 Sensitivity analyses

To strengthen the evidence of causal relationships, sensitivity analyses were conducted under different assumptions. Consistency across estimates strengthened the evidence of causal relationships. Sensitivity analyses included weighted median (15) (assuming majority validity), robust adjusted profile score (assuming a normal distribution of pleiotropic effects close to zero), MR PRESSO (testing robustness), and MR Egger (16) (assuming instrument strength is independent of direct effects). A significant MR Egger intercept (P<0.05) indicates a potential violation of the exclusion restriction hypothesis through overall level pleiotropy.

To further mitigate pleiotropic effects of the instruments, we employed PhenoScanner, a carefully curated genotype-phenotype cross-referencing tool, to identify any pleiotropic effects of the genetic instruments. In sensitivity analysis, we excluded genetic variants [rs1800562 in HFE and rs855791 in TMPRSS6 which relate to blood group, hereditary hemochromatosis and iron deficient anemia (17, 18)] known to be directly related to phenotypes associated with bone and joint-related diseases.

### 2.5 Reverse causality analysis

In order to explore the potential reverse causal relationship between iron status and bone and joint-related diseases, we conducted a reverse causality analysis. Given the plausible causal relationship between iron status and these diseases, we reversed the exposure and outcomes. Standard procedures were followed to select appropriate genetic tools, and the IVW method was used for primary analysis. Sensitivity analysis was also performed to evaluate the robustness of our findings.

All analyses were conducted using R version 4.3.0 and R software packages [“TwoSampleMR” 0.5.6 (19) and “MRPRESSO” version 1.0 (20)].

## 3 Results

The SNPs we identified exhibited a significant correlation with serum iron status according to conventional GWAS thresholds (P<5 × 10^-8^). To cluster these SNPs, we estimated linkage disequilibrium (LD) using data from the 1,000 Genome Project samples of European origin. We set an LD threshold of 0.1 and a window size of 10,000kb. Within SNP pairs showing LD estimates >0.1, only SNPs with lower P values were retained. We removed SNPs with secondary allele frequencies <1%. The main analysis included 22 serum iron SNPs, 5 ferritin SNPs, 36 TSAT SNPs, and 41 transferrin SNPs after excluding unsuitable genetic tools. The overall F-scores were as follows: serum iron - 89 (R^2^:3.7%), ferritin - 60 (R^2^:2.5%), TSAT - 107 (R^2^:4.4%), and transferrin - 143 (R^2^:5.9%), indicating that weak instrument bias was unlikely to be present. **Supplementary Tables S3**-**S5** provides a list of all selected SNPs along with their relevant data for subsequent causal analysis, including β (b), standard error (SE), effect alleles/other alleles, p-values, R^2^, and F-scores. Models using regression analysis were employed to investigate the effect size (b) of each SNP in relation to traits. Linear regression was used for quantitative traits (each iron state), while logistic regression was used for qualitative outcome traits, assuming if there is a linear trend in each copy of an allele. The regression coefficients of quantitative traits (each iron state) are represented by a higher standard deviation per unit, while the regression coefficients of qualitative outcome traits are represented by larger logarithmic advantages per unit, enabling comparisons across various traits. The F-statistic for all SNPs exceeded 10, indicating a strong correlation between instrumental variables and exposure features.

**Figure 2:**
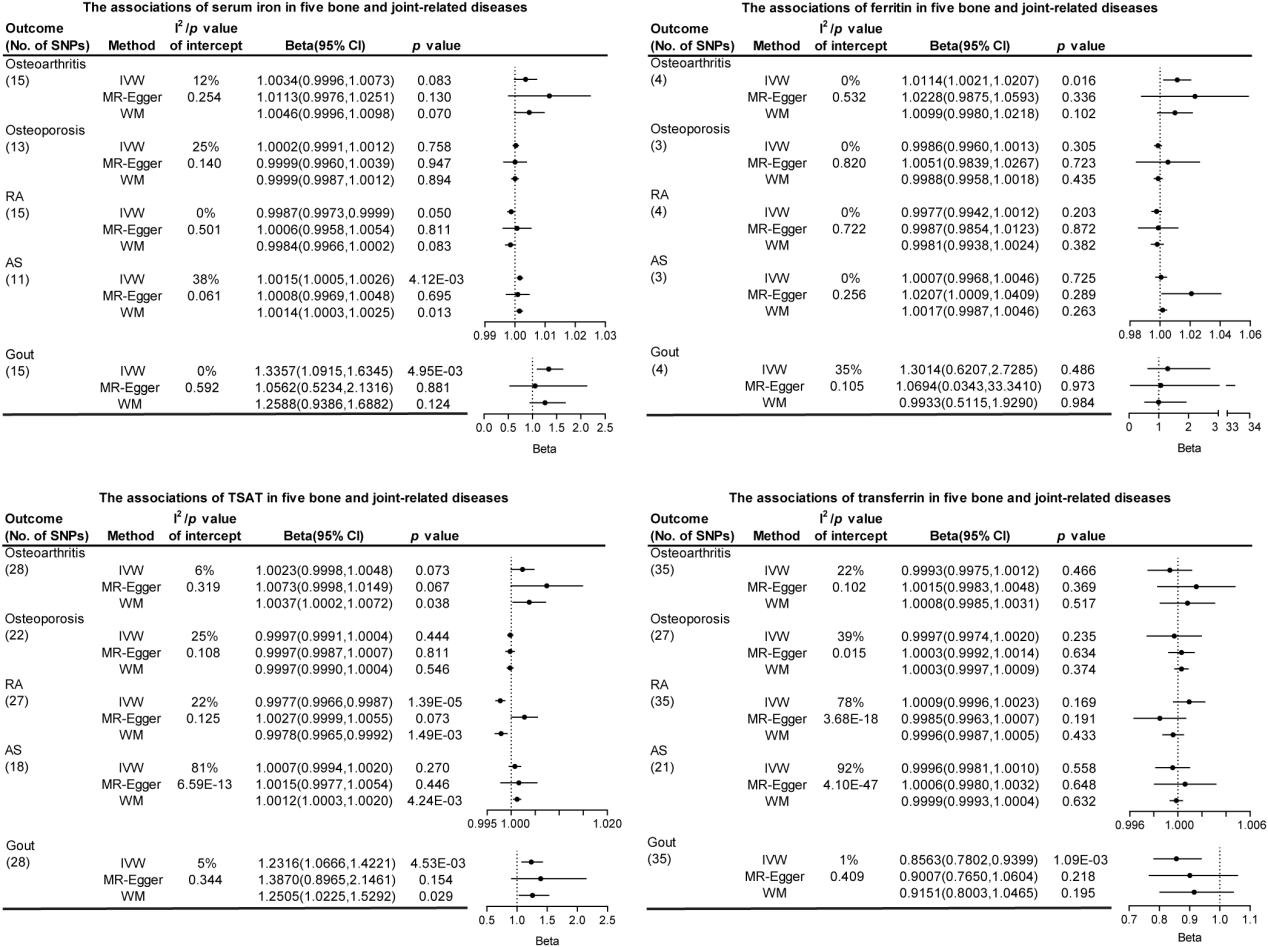
The association of iron status in the risk of five bone and joint-related diseases. SNPs, single nucleotide polymorphisms; I^2^, degree of heterogeneity; CI, confidence interval; IVW, inverse variance weighting; WM, weighted median; TSAT, transferrin saturation; RA, rheumatoid arthritis; AS, ankylosing spondylitis.

**Supplementary Tables S6**-**S7** presents the preliminary analysis results of the impact of gene-predicted iron status on the risk of different bone and joint-related diseases. For osteoarthritis, ferritin levels were positively correlated with the risk (OR [95% CI], 1.0114 [1.0021-1.0207]), while other iron states showed no causal relationship. For RA, there was a negative correlation between serum iron and TSAT levels predicted by genes and their risk (OR [95% CI], 0.9987 [0.9973-0.9999] and 0.9977 [0.9966-0.9987], respectively), while no causal relationship existed between ferritin and transferrin. Regarding AS, serum iron levels were positively correlated with the risk (OR [95% CI], 1.0015 [1.0005-1.0026]), while other iron states showed no causal relationship. For gout, gene-predicted serum iron and TSAT exhibited a positive correlation with the risk (OR [95% CI], 1.3357 [1.0915-1.6345] and 1.2316 [1.0666-1.4221], respectively), whereas transferrin showed a negative correlation (OR [95% CI], 0.8563 [0.7802-0.9399]). No causal relationship was found between iron status predicted by all genes and the risk of osteoporosis (**Figure 2**; **Supplementary Figures 1**-**5**).

**Figure 3:**
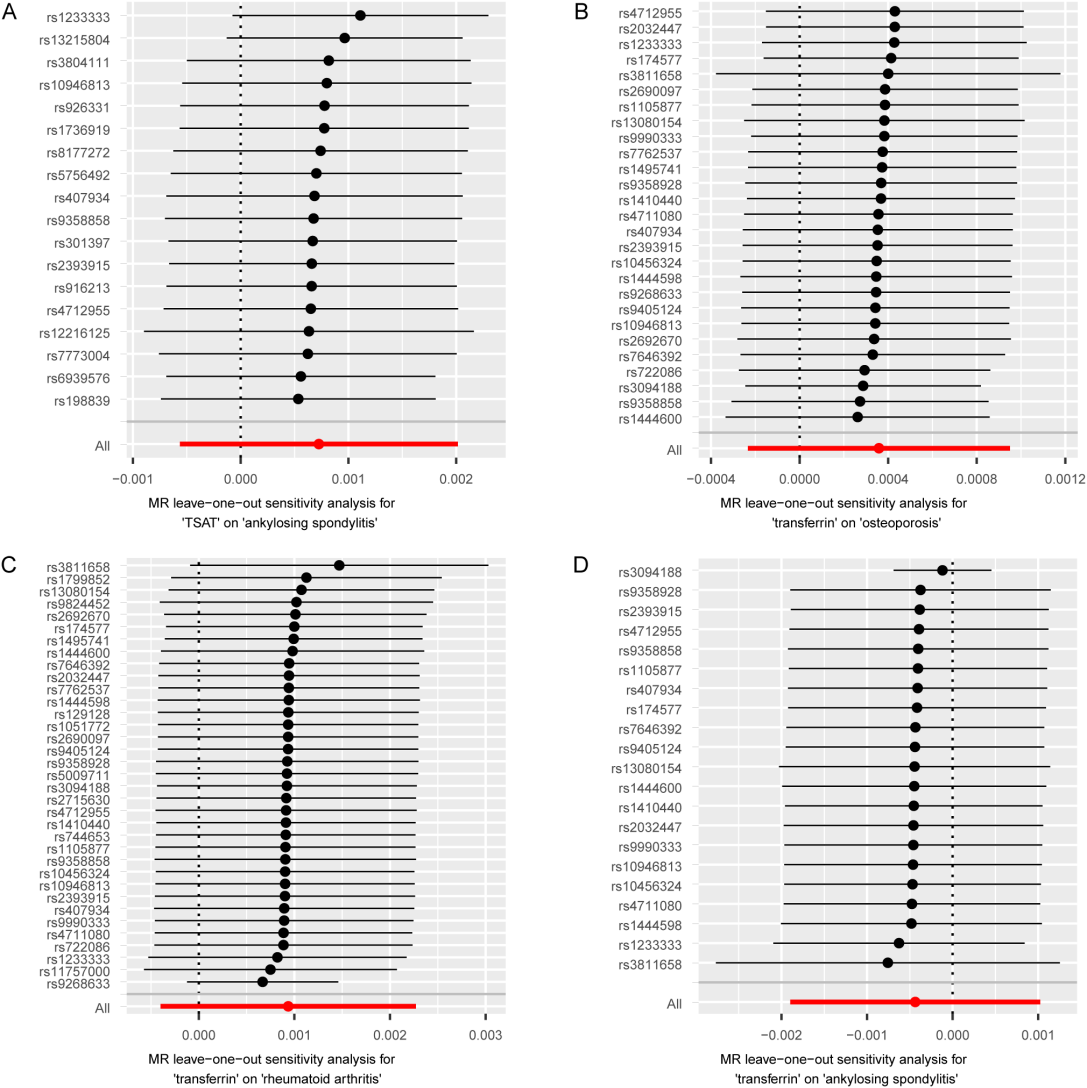
MR leave-one-out sensitivity analysis of serum iron status on bone and joint-related diseases. Circles indicate the results of MR analysis of remaining SNPs on serum iron status on bone and joint-related diseases after omitting each SNP in turn. Bars indicate CI. **(A)** Leave-one-out plot from TSAT on AS. **(B)** Leave-one-out plot from transferrin on osteoporosis. **(C)** Leave-one-out plot from transferrin on RA. **(D)** Leave-one-out plot from transferrin on AS. MR, Mendelian randomization; SNPs, single nucleotide polymorphisms; CI, confidence interval; TSAT, transferrin saturation; RA, rheumatoid arthritis; AS, ankylosing spondylitis.

To mitigate potential bias, we performed a series of sensitivity analyses to assess the reliability of the MR analysis and identify any potential pleiotropy. The results can be found in **Supplementary Table S7**. In the MR Egger regression analysis, horizontal pleiotropy was observed between TSAT and RA (p= 0.001), as well as transferrin and RA (p=0.01). Next, their horizontal pleiotropy was detected using the MR PRESSO package. The results revealed no evidence of horizontal pleiotropy between TSAT and RA (p=0.592) or transferrin and RA (p=0.114). Cochran’s Q-test indicated heterogeneity between TSAT and AS, as well as transferrin and osteoporosis, transferrin and RA, transferrin and AS. However, in the leave-one-out analysis, removing any SNP did not alter the basic causal effect, demonstrating the robustness of our MR analysis (**Figure 3**).

**Figure 4:**
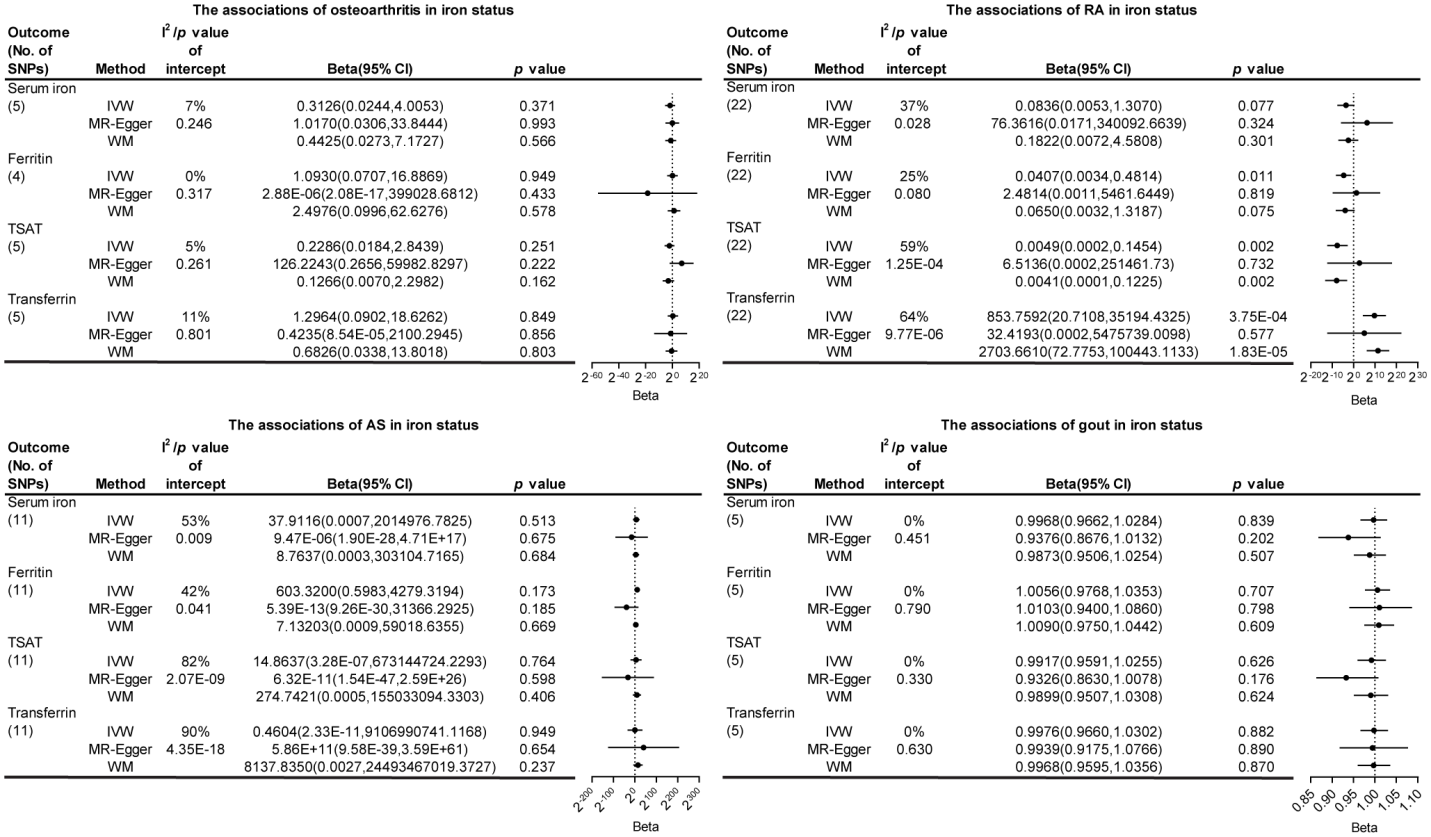
The association of bone and joint-related diseases in iron status. SNPs, single nucleotide polymorphisms; I^2^, degree of heterogeneity; CI, confidence interval; IVW, inverse variance weighting; WM, weighted median; TSAT, transferrin saturation; RA, rheumatoid arthritis; AS, ankylosing spondylitis.

**Figure 5:**
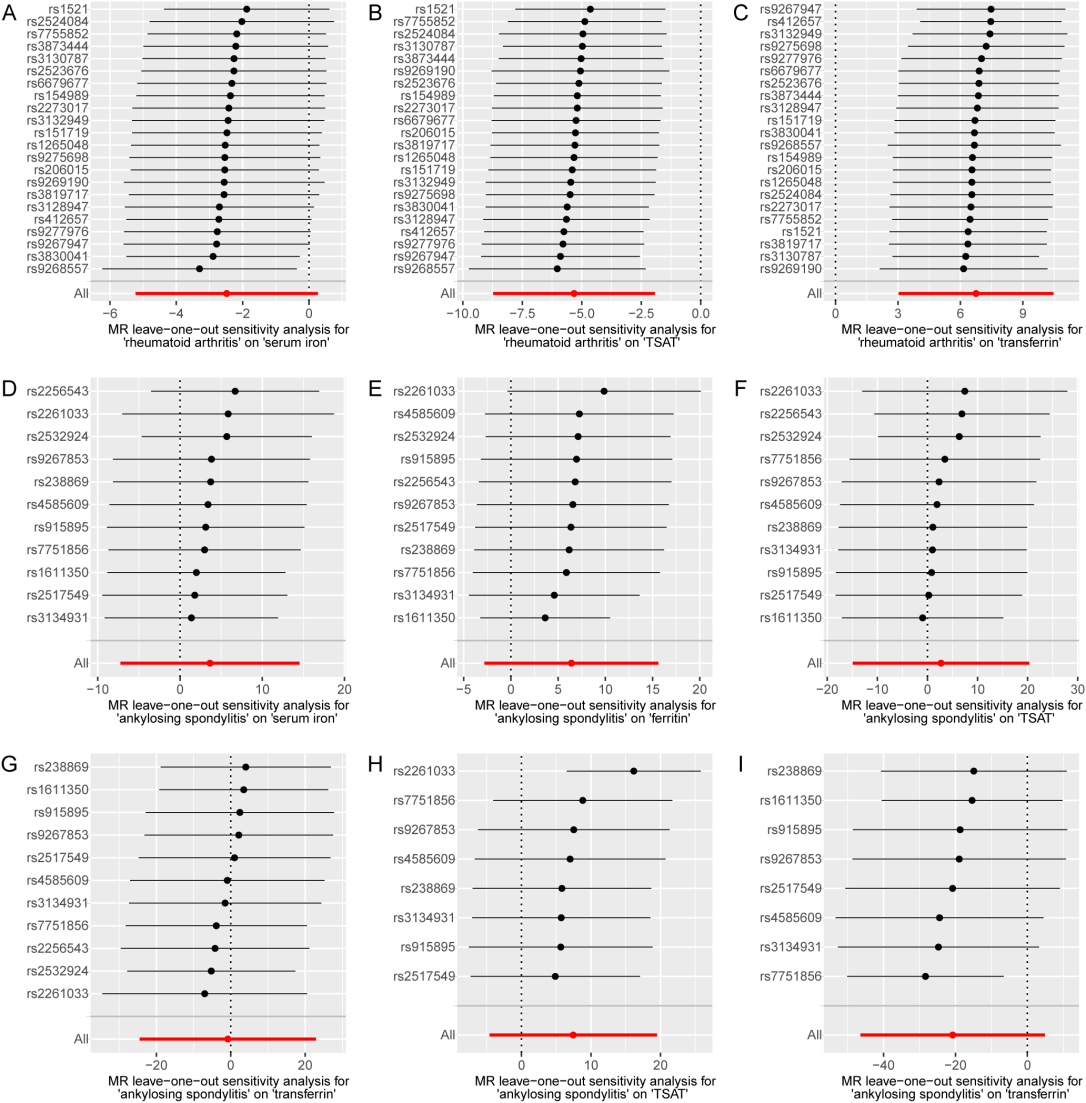
MR leave-one-out sensitivity analysis of bone and joint-related diseases on serum iron status. Circles indicate the results of MR analysis of remaining SNPs on bone and joint-related diseases on serum iron status after omitting each SNP in turn. Bars indicate CI. **(A)** Leave-one-out plot from RA on serum iron. **(B)** Leave-one-out plot from RA on TSAT. **(C)** Leave-one-out plot from RA on transferrin. **(D)** Leave-one-out plot from AS on serum iron. **(E)** Leave-one-out plot from AS on ferritin. **(F)** Leave-one-out plot from AS on TSAT. **(G)** Leave-one-out plot from AS on transferrin. **(H)** Leave-one-out plot from AS on TSAT after detecting three SNPs. **(I)** Leave-one-out plot from AS on transferrin after detecting three SNPs. MR, Mendelian randomization; SNPs, single nucleotide polymorphisms; CI, confidence interval; TSAT, transferrin saturation; RA, rheumatoid arthritis; AS, ankylosing spondylitis.

In the reverse causality analysis, we conducted IVW analysis to explore the potential causal effects of bone and joint-related diseases on iron status (**Figure 4**; **Supplementary Figures 6**-**9**; **Supplementary Tables S8**-**S10**). The results indicated that RA had a causal effect on ferritin, TSAT, and transferrin levels. Specifically, RA showed a negative correlation with ferritin and TSAT levels, and a positive correlation with transferrin levels. The ORs and their corresponding 95% CIs are as follows: ferritin (OR: 0.0407, 95% CI: 0.0034 to 0.4814, p=0.01), TSAT (OR: 0.0049, 95% CI: 0.0002 to 0.1454, p=2.13 × 10^-3^), and transferrin (OR: 853.76, 95% CI: 20.71 to 35,194.43, p=3.75 × 10^-4^). Additionally, we conducted MR Egger regression analysis to evaluate targeted pleiotropy. The intercept of the MR Egger regression revealed no evidence of directed pleiotropy in any of the causal relationship estimates (p>0.05). Cochran’s Q-test indicated significant heterogeneity between RA and iron status (except for RA and ferritin), as well as AS and iron status. The leave-one-out analysis conducted between AS and TSAT detected three SNPs (rs1611350, rs2256543 and rs2532924) that would drive the result and make the causal estimate unreliable, as well as three SNPs (rs2261033, rs2532924 and rs2256543) between AS and transferrin. In the other leave-one-out analyses, removing any SNP did not alter the basic causal effect, demonstrating the robustness of our MR analysis (**Figure 5**). **Supplementary Tables S11**-**S12** shows the analysis results of the impact of gene-predicted AS on the risk of TSAT and transferrin after removing the relevant SNPs mentioned above. The results show no causal relationship between genetic prediction of AS and the risk of TSAT or transferrin.

## 4 Discussion

In this study, we utilized validated structured MR analysis to estimate the causal relationship between iron status and the risk of bone and joint-related diseases. Our findings revealed a causal link between ferritin and an increased risk of osteoarthritis, a causal association between serum iron and TSAT levels and a reduced risk of RA, a causal association between serum iron and an increased risk of AS, a causal relationship between serum iron and TSAT levels and an elevated risk of gout, and a causal correlation between transferrin and a decreased risk of gout. However, no causal relationship was observed between iron status and osteoporosis in our study. These results remained consistent in the main MR analysis as well as subsequent sensitivity analyses. These findings emphasize the potential of iron status as a biomarker for specific bone and joint-related diseases.

Iron is an essential metal that serves as a cofactor in various chemical reactions crucial for cell survival, growth, and metabolism (21). However, excessive iron under aerobic conditions can lead to oxidative stress, inflammation and tissue damage. Previous epidemiological and experimental studies have reported the association between serum iron status and osteoarthritis. Numerous studies have indicated that iron overload can induce oxidative stress damage and ferroptosis in chondrocytes, contributing to the onset and progression of osteoarthritis (22–24). Glutathioneperoxidase4 (GPX4) is a key regulator of ferroptosis, and its downregulation can increase the sensitivity of chondrocytes to oxidative stress and aggravate extracellular matrix degradation through the MAPK/NF-κB pathway (25). A previous MR study on iron status and osteoarthritis demonstrated a positive correlation between TSAT and osteoarthritis, while transferrin exhibited a negative correlation (8). Our study identified a positive causal relationship between ferritin and the risk of osteoarthritis. This discrepancy could be attributed to the previous study’s use of a relatively small number of genetic tools and the inclusion of a variant associated with hereditary hemochromatosis (rs1800562 in HFE), which has significant pleiotropic effects (17). Hence, the observed research outcomes may be influenced by hereditary hemochromatosis rather than iron status.

Several studies have emphasized the importance of iron in osteoporosis. Maurer et al. reported a positive association between dietary iron intake and bone density in women receiving hormone replacement therapy (26). Similarly, Okyay et al. found that low serum iron levels appear to be a risk factor for postmenopausal women developing osteoporosis (27). Zhao et al. discovered that mild iron deficiency can promote osteoblast activity, while severe iron deficiency inhibits bone formation (28). However, a MR study on iron status and osteoporosis revealed no genetic causal relationship between iron status and the risk of osteoporosis (29), which aligns with our findings.

The association between iron status and the risk of RA has yielded contradictory findings. Two previous cohort studies reported no significant relationship between dietary iron intake and the risk of RA (3, 30). Similarly, a clinical study found no notable difference in ferritin levels between individuals with and without RA (31). In our MR study, we observed a negative correlation between serum iron and TSAT levels and the risk of RA. The conclusions are consistent with some studies showing iron deficiency in the blood and iron accumulation in the synovium and synovial fluid in patients with rheumatoid arthritis, which may indicate iron redistribution (32). However, these results may be influenced by residual confounding and inherent defects in estimates from observational studies. Additionally, slightly higher dietary iron intake may not significantly impact serum iron content. Further comprehensive research is necessary to elucidate the mechanisms underlying the protective effect of high-speed rail levels on RA.

Limited data are available regarding the relationship between iron status and the risk of AS. A case-control study involving 30 patients with AS demonstrated significantly higher average iron intake and iron accumulation compared to the control group (33). However, observational studies are susceptible to confounding factors and reverse causal relationships, leading to non-causal associations. Therefore, our MR analysis revealed a positive correlation between serum iron and the risk of AS.

Multiple studies have explored the link between iron status and gout. A randomized controlled multicenter clinical study identified a positive correlation between serum ferritin levels and the risk of gout (2). Another large community cohort study suggested that iron deficiency anemia is associated with an increased risk of gout (34), indicating that pathologically low iron status can also impact gout development. Consistent with these findings, our MR analysis demonstrated a positive correlation between serum iron and TSAT levels and the risk of gout, while transferrin exhibited a negative correlation with the risk of gout development.

The findings from the reverse causal relationship analysis suggest a negative correlation between RA and ferritin and TSAT, while a positive correlation existed between RA and transferrin levels. However, there was no reverse causal relationship observed between osteoarthritis, AS, gout and iron status. These consistent results were confirmed in subsequent sensitivity analyses. A previous clinical study involving 62 patients with RA indicated that over 60% of patients exhibited iron deficiency, characterized by lower ferritin and transferrin levels (35). Nevertheless, genetic studies investigating the impact of RA on iron status have been rarely reported. Our study revealed a strong negative correlation between RA and ferritin and TSAT levels, as well as a significant positive correlation with transferrin levels. These findings suggest that iron status may serve as key indicators of RA progression. However, further direct research is needed to validate these indirect findings and may help partially explain the reverse causal relationship between RA and iron status.

Our research had some limitations. The disease GWAS data we utilized were obtained from publicly available sources, limiting our ability to perform subgroup analyses considering factors such as age and gender. Furthermore, genetic variations identified through GWAS research may differ across various ethnic backgrounds, potentially reducing the generalizability of our results targeting the European population to other genetic backgrounds. Therefore, further extensive research is necessary for diverse populations. Additionally, Cochran’s Q-test revealed significant heterogeneity in certain data points in our study. However, MR-PRESSO analysis excluded horizontal pleiotropy, indicating that heterogeneity did not introduce bias or pleiotropy in our study.

## 5 Conclusion

To the best of our knowledge, this was the first comprehensive study utilizing MR analysis based on publicly available genetic data to investigate the causal relationship between iron status and bone and joint-related diseases. Our study demonstrated that elevated serum iron and TSAT levels increased the risk of gout, while appropriately high serum iron and TSAT levels might provide a protective effect against RA. Different iron states exhibit varying roles in regulating bone and joint-related diseases, necessitating further in-depth research to elucidate the underlying mechanisms. For patients with bone and joint-related diseases, routine monitoring of iron status may play a crucial role in their treatment. Abnormal iron status should be screened for early detection and treatment of bone and joint-related diseases. Our research findings may provide a new strategy for understanding the relationship between iron status and bones.

## Data Availability

The original contributions presented in the study are included in the article/**Supplementary Material**. Further inquiries can be directed to the corresponding author.
