# Supplementary material for: Association between serum iron status and the risk of five bone and joint-related diseases: a Mendelian randomization analysis
Source: Front Endocrinol (Lausanne). 2024 Sep 13;15:1364375. doi: 10.3389/fendo.2024.1364375 (PMC11427247; doi:10.3389/fendo.2024.1364375)
Supplement: Supplementary file 1 [file DataSheet1.docx]

Supplementary Material

# Supplementary Figures

**Supplementary Figure 1.** Forest plot of causal effect of iron status on osteoarthritis.

**Supplementary Figure 2.** Forest plot of causal effect of iron status on osteoporosis.

**Supplementary Figure 3.** Forest plot of causal effect of iron status on rheumatoid arthritis.

**Supplementary Figure 4.** Forest plot of causal effect of iron status on ankylosing spondylitis.

**Supplementary Figure 5.** Forest plot of causal effect of iron status on gout.

**Supplementary Figure 6.** Forest plot of causal effect of bone and joint-related diseases on serum iron.

**Supplementary Figure 7.** Forest plot of causal effect of bone and joint-related diseases on ferritin.

**Supplementary Figure 8.** Forest plot of causal effect of bone and joint-related diseases on TSAT.

**Supplementary Figure 9.** Forest plot of causal effect of bone and joint-related diseases on transferrin.

# Supplementary Figure 1


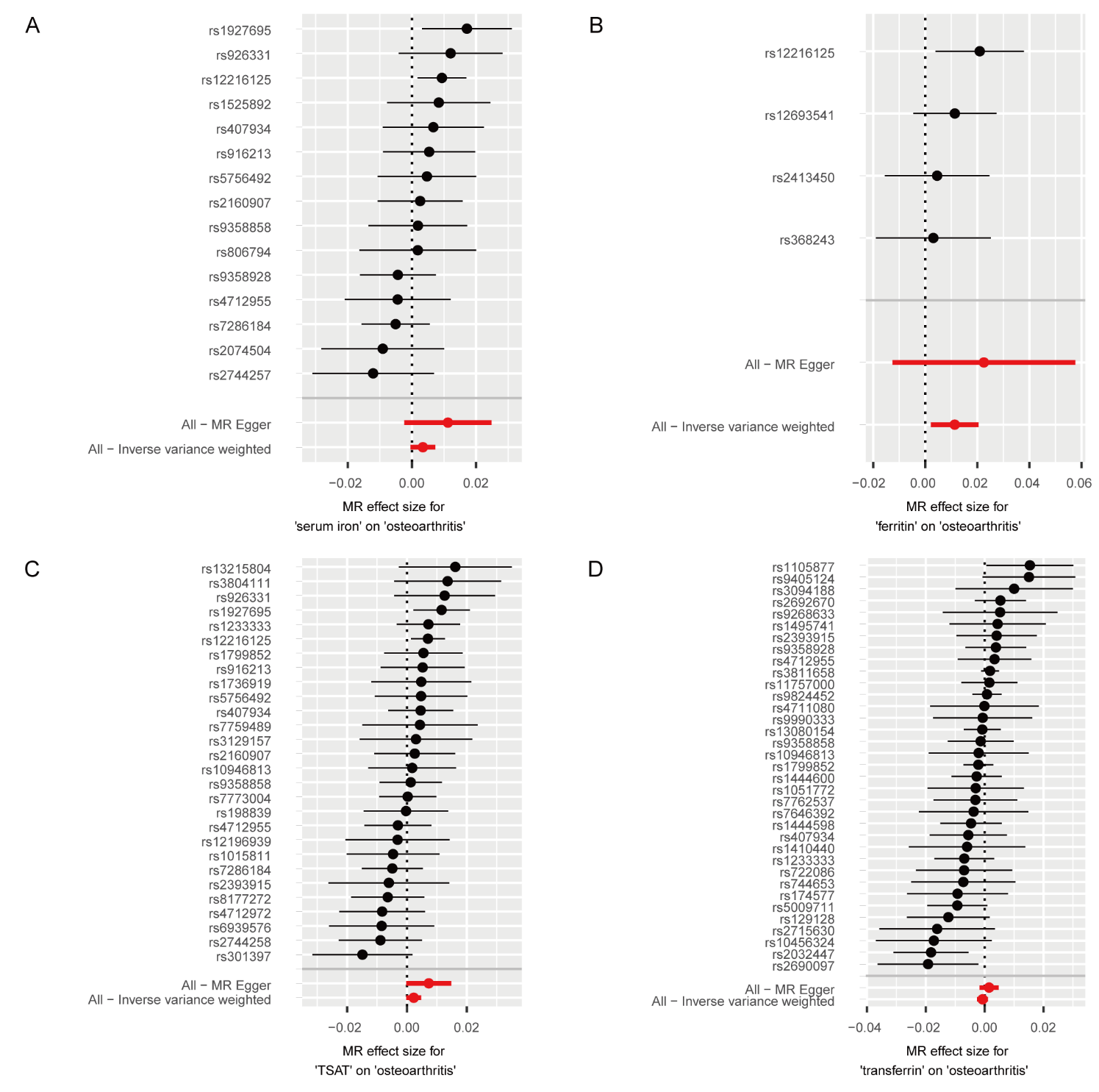


**Supplementary Figure 1.** Forest plot of causal effect of iron status on osteoarthritis. (A) serum iron. (B) ferritin. (C) TSAT. (D) transferrin.

# Supplementary Figure 2

**
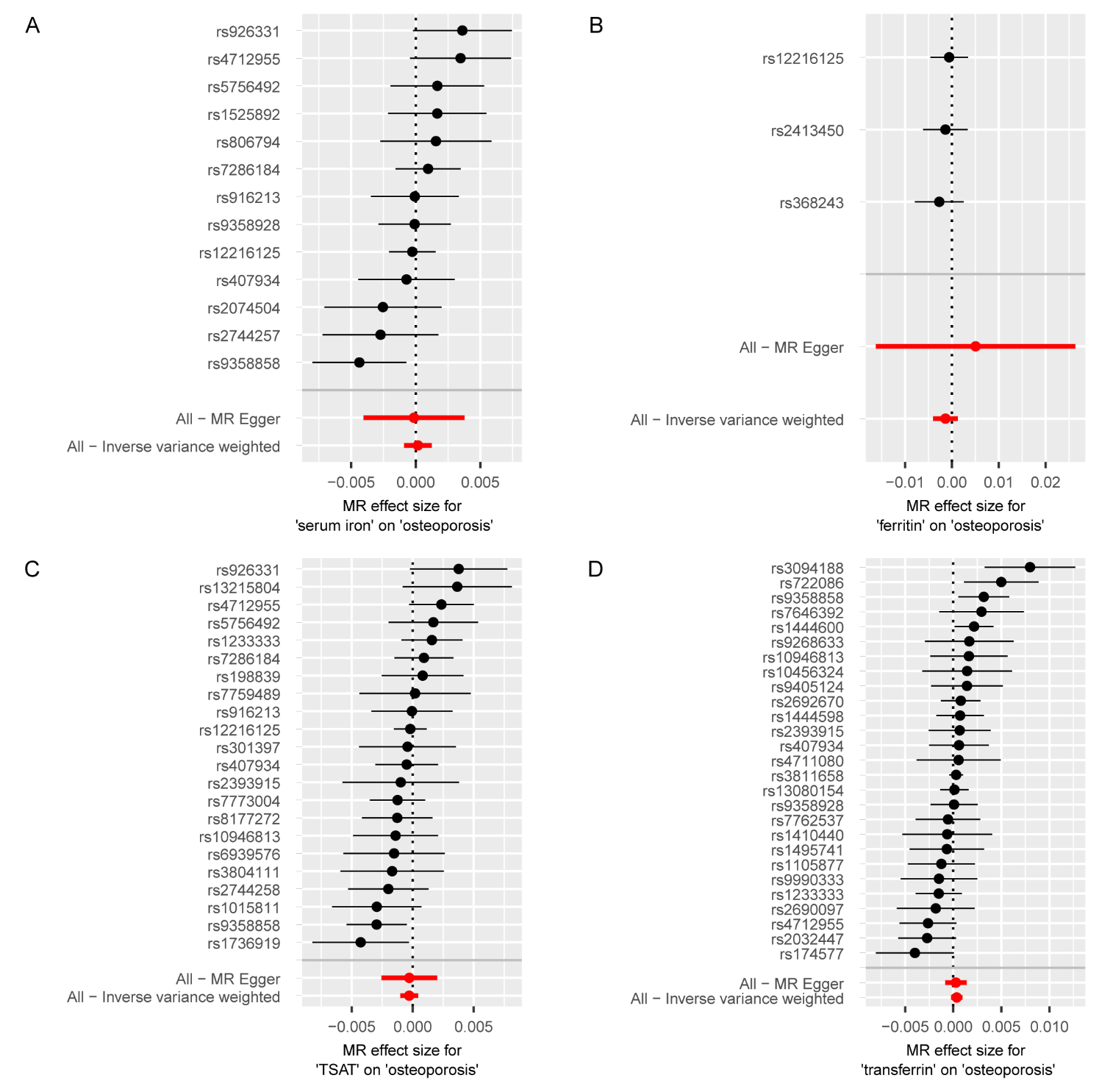
**

**Supplementary Figure 2.** Forest plot of causal effect of iron status on osteoporosis. (A) serum iron. (B) ferritin. (C) TSAT. (D) transferrin.

# Supplementary Figure 3
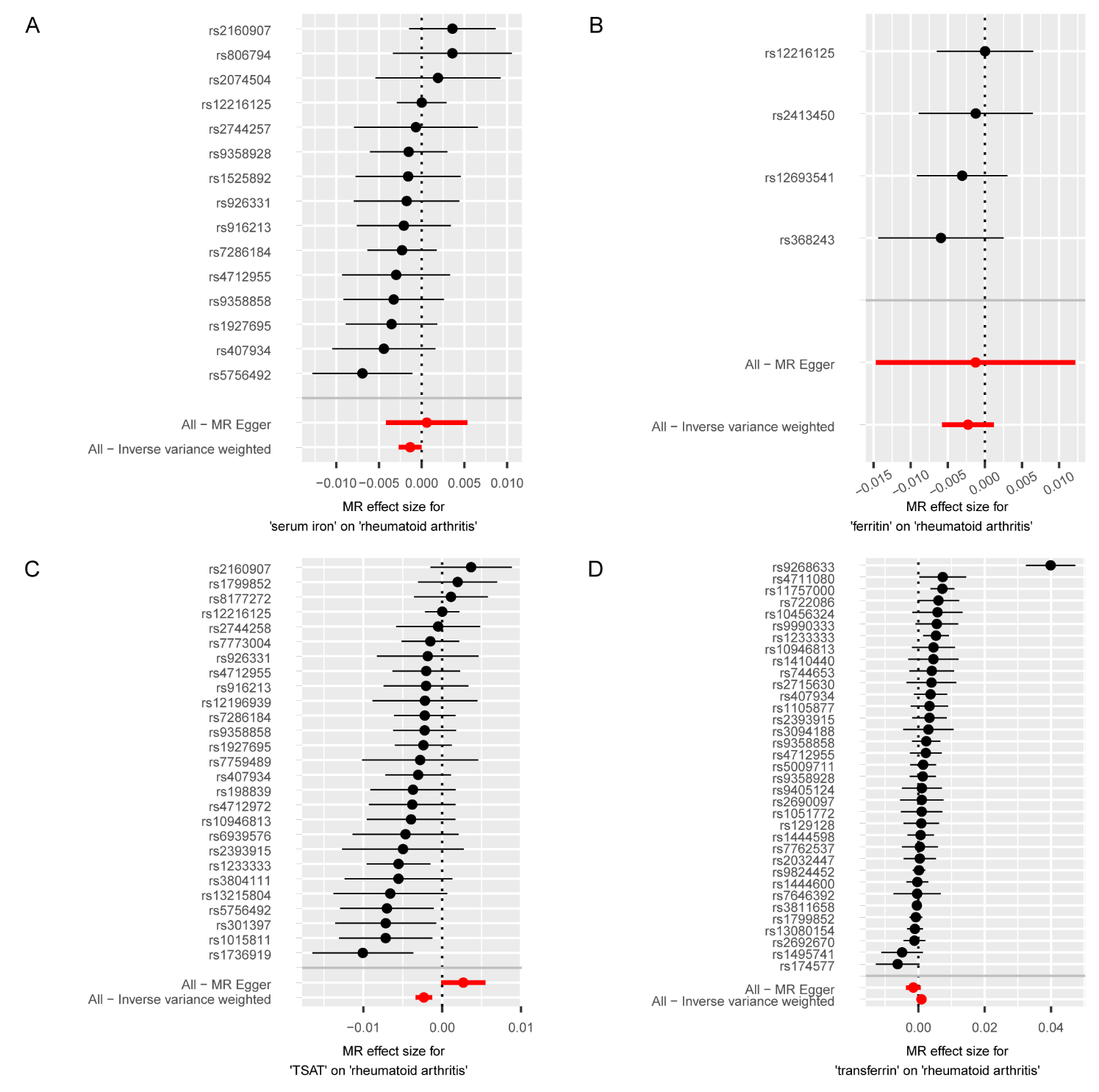


**Supplementary Figure 3.** Forest plot of causal effect of iron status on rheumatoid arthritis. (A) serum iron. (B) ferritin. (C) TSAT. (D) transferrin.

# Supplementary Figure 4


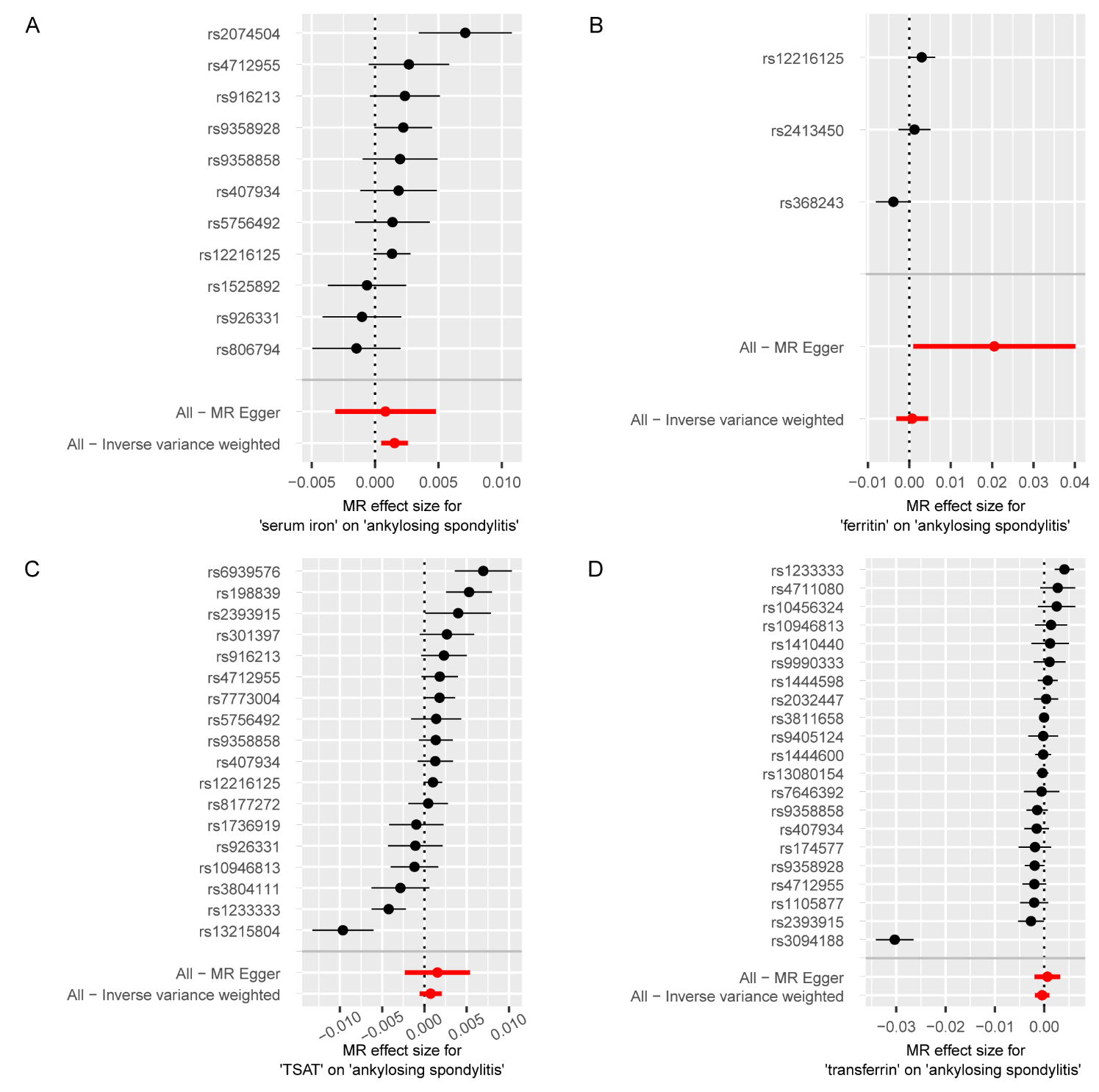


**Supplementary Figure 4.** Forest plot of causal effect of iron status on ankylosing spondylitis. (A) serum iron. (B) ferritin. (C) TSAT. (D) transferrin.

# Supplementary Figure 5


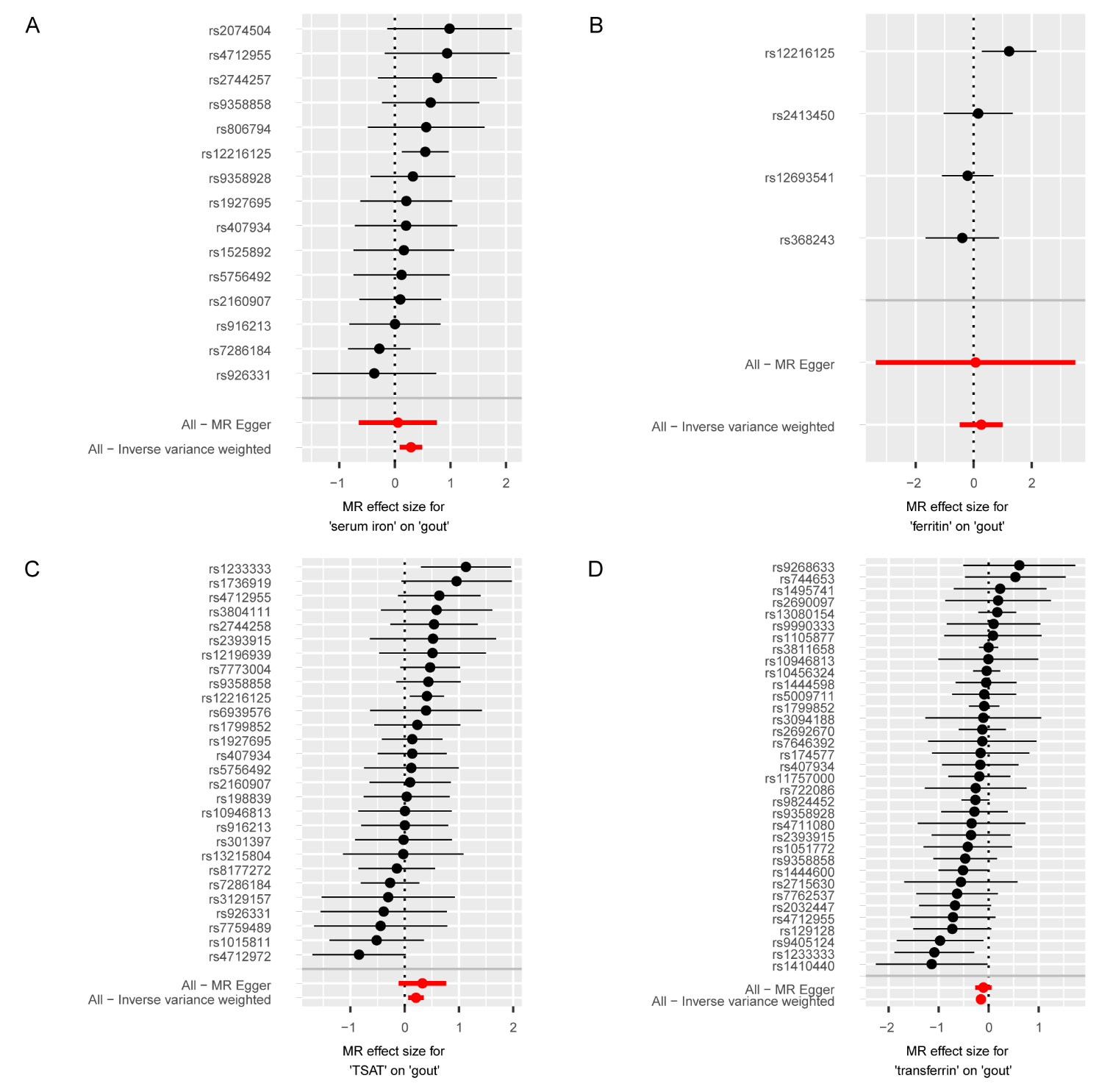


**Supplementary Figure 5.** Forest plot of causal effect of iron status on gout. (A) serum iron. (B) ferritin. (C) TSAT. (D) transferrin.

# Supplementary Figure 6


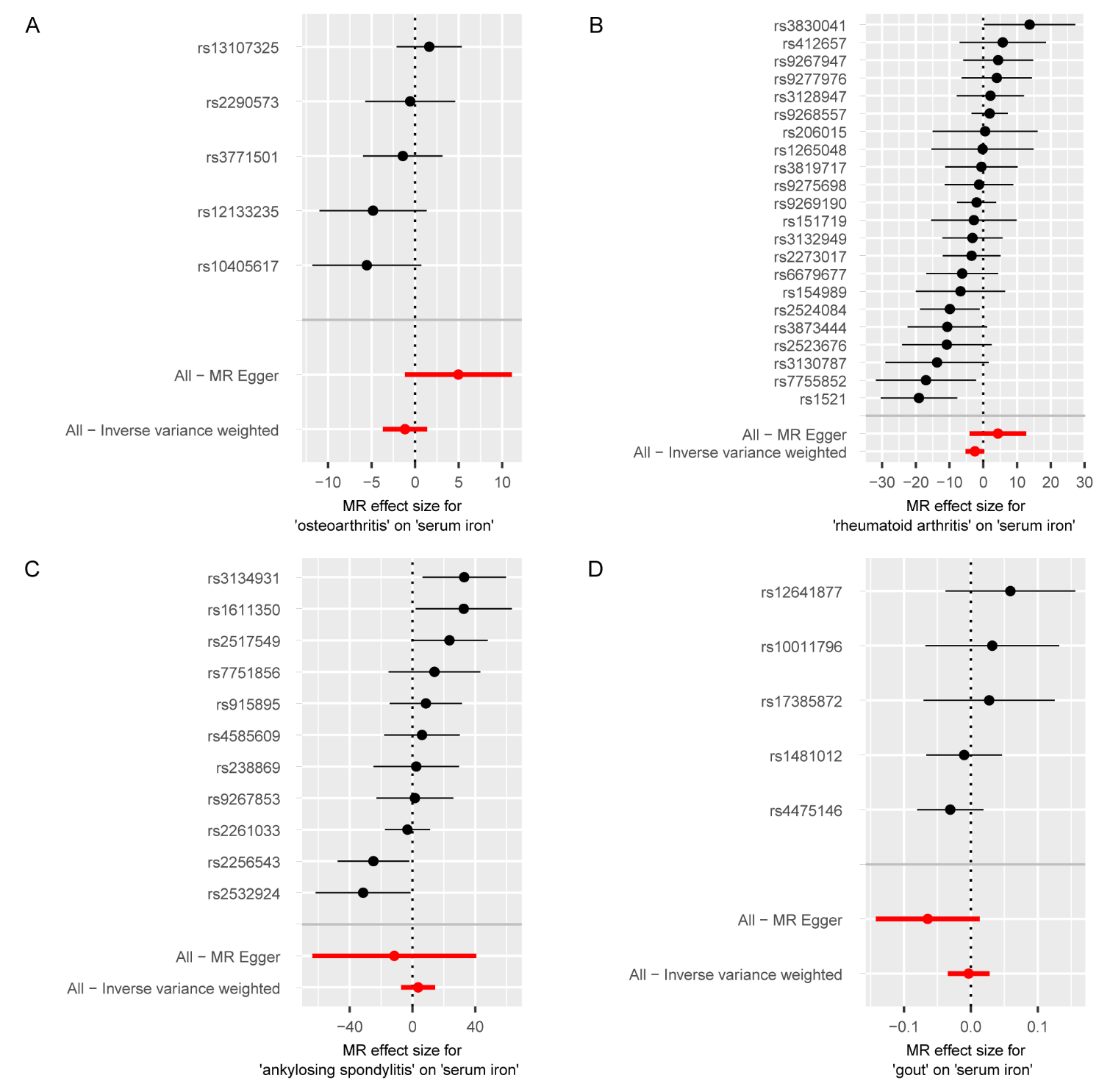


**Supplementary Figure 6.** Forest plot of causal effect of bone and joint-related diseases on serum iron. (A) osteoarthritis. (B) rheumatoid arthritis. (C) ankylosing spondylitis. (D) gout.

# Supplementary Figure 7


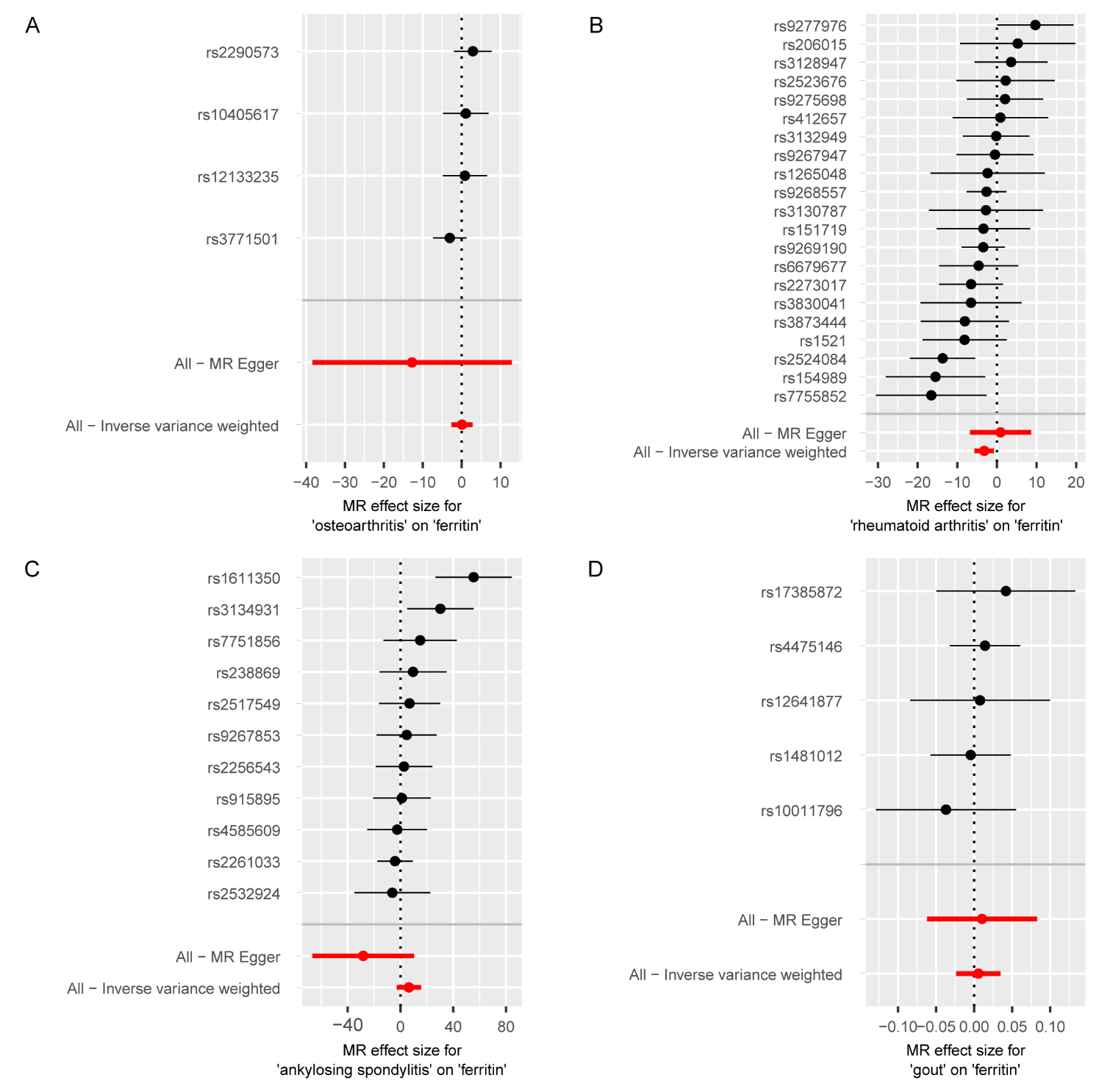


**Supplementary Figure 7.** Forest plot of causal effect of bone and joint-related diseases on ferritin. (A) osteoarthritis. (B) rheumatoid arthritis. (C) ankylosing spondylitis. (D) gout.

# Supplementary Figure 8


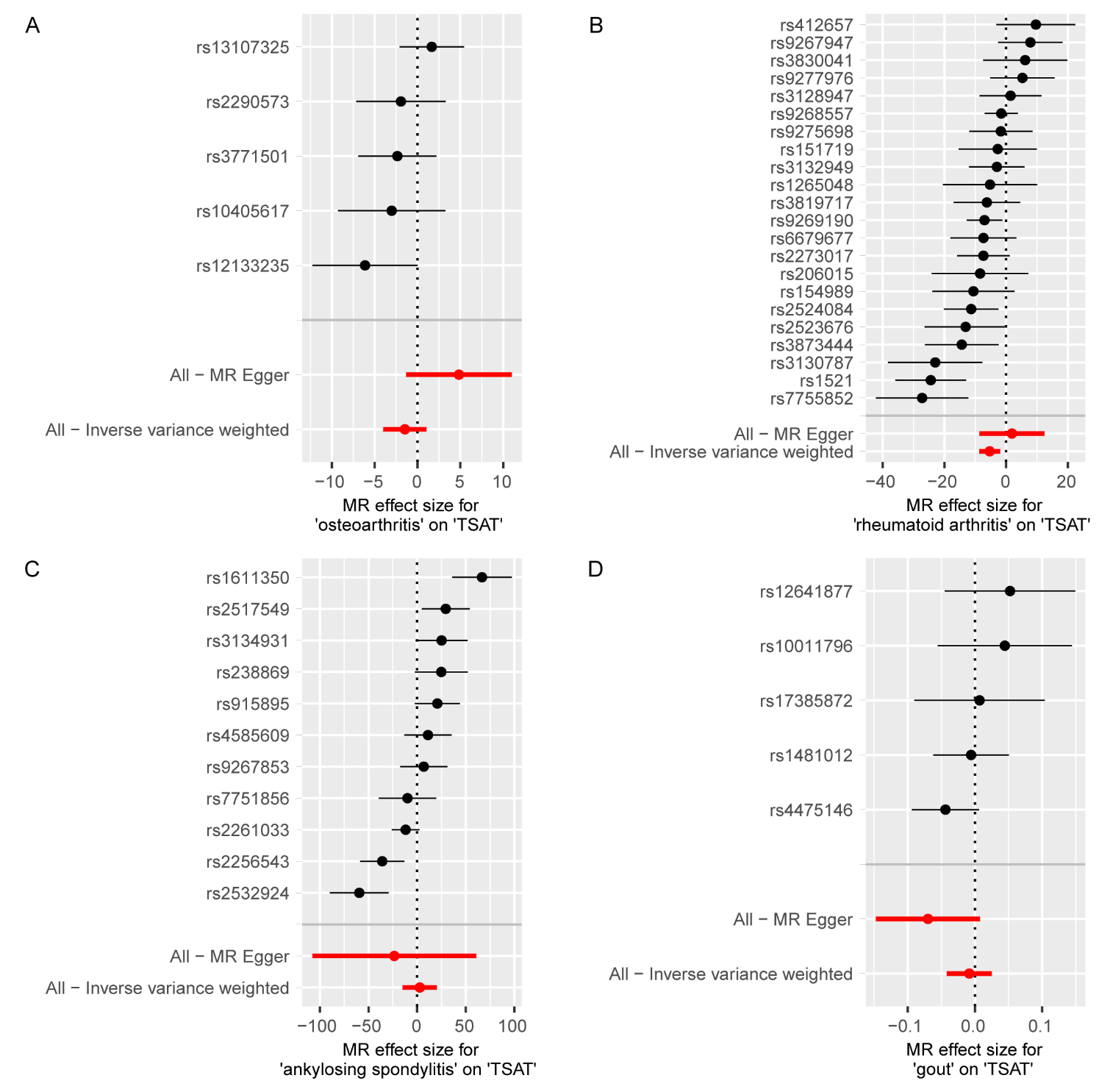


**Supplementary Figure 8.** Forest plot of causal effect of bone and joint-related diseases on TSAT. (A) osteoarthritis. (B) rheumatoid arthritis. (C) ankylosing spondylitis. (D) gout.

# Supplementary Figure 9


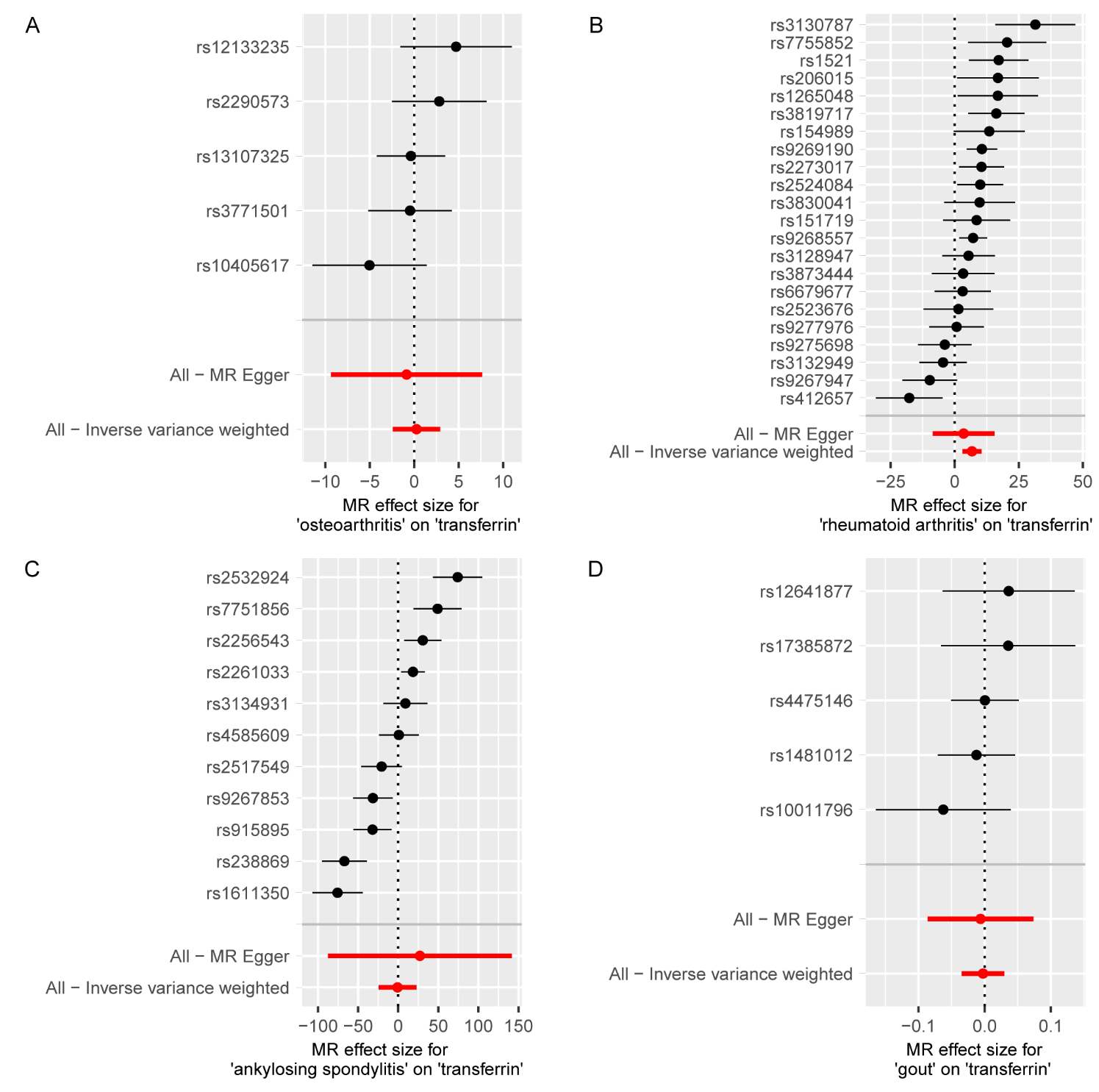


**Supplementary Figure 9.** Forest plot of causal effect of bone and joint-related diseases on transferrin. (A) osteoarthritis. (B) rheumatoid arthritis. (C) ankylosing spondylitis. (D) gout.
